# Comparison of Two Tubularized Incised Plate Urethroplasty Techniques in Hypospadias Reconstructive Surgery

**DOI:** 10.29252/wjps.9.3.254

**Published:** 2020-09

**Authors:** Katerina Kambouri, Maria Aggelidou, Savvas Deftereos, Christos Tsalikidis, Pelagia Chloropoulou, Sotirios Botaitis, Stelios Giannakopoulos, Michail Pitiakoudis

**Affiliations:** 1 Department of Anesthesiology, Department of Pediatric Surgery, Alexandroupolis University Hospital, Democritus University of Thrace School of Medicine, Alexandroupolis, Greece;; 2 Department of Radiology, Alexandroupolis University Hospital, Democritus University of Thrace School of Medicine, Alexandroupolis, Greece;; 3 Department of Surgery, Alexandroupolis University Hospital, Democritus University of Thrace School of Medicine, Alexandroupolis, Greece;; 4 Anesthesiology, Alexandroupolis University Hospital, Democritus University of Thrace School of Medicine, Alexandroupolis, Greece;; 5 Department of Urology, Alexandroupolis University Hospital, Democritus University of Thrace School of Medicine, Alexandroupolis, Greece

**Keywords:** Hypospadias, Urethroplasty, Operation, Children

## Abstract

**BACKGROUND:**

Hypospadias repair is a challenging type of urogenital reconstructive surgery for which different techniques are currently used. The purpose of this study is to determine the outcomes of distal, mid-shaft and proximal hypospadias repair using two new variations of tubularized incised plate (TIP) urethroplasty (TIP-δ and TIP-ελ) and to compare their complication rates with other already known operative techniques made from the same surgical team.

**METHODS:**

This study included 269 boys with hypospadias. The preoperative meatal site was distal in 179 patients, mid-shaft in 44 and proximal in 46. The average age at the operation was 17 months. The technique applied in distal hypospadias was Mathieu in 77 patients, Snodgrass in 28 and (TIP)-δ in 74. The technique applied in mid-shaft hypospadias was a tubularized island flap (TIF) in 12 patients, onlay island flap (OIF) in 5 and TIP-ελ in 27. The operative technique for proximal hypospadias was TIF in 15 patients, OIF in 10 and TIP-ελ in 21. TIP-δ and TIP-ελ are two new variants of TIP operation that we have used in our clinic since 2010. Postoperative complications were recorded, and we compared the outcomes obtained by applying the techniques.

**RESULTS:**

The use of TIP-δ in the distal hypospadias and long TIP-ελ in the mid-shaft and proximal hypospadias resulted in significantly fewer complications than the other surgical methods across all cases of hypospadias (*p*<0.05).

**CONCLUSION:**

The type of tissue used for neourethral coverage seems to play an important role in the outcome of hypospadias surgery.

## INTRODUCTION

Hypospadias is a congenital abnormality that occurs in 1 of 200 live births in which the urethral meatus lies ectopically on the ventral surface of the penis, from just below the tip of the glans to the perineum in the most-severe cases.^1^^-^^3^ The purpose of hypospadias repair is to construct a urethra that enables the patient to urinate adequately and to produce a penis with a satisfactory cosmetic result. Different techniques for hypospadias repair have been described and newer methods continue to evolve. There is no standard procedure for all types of hypospadias repairs.^4^

Several factors interact to determine the optimal type of repair, such as the meatal site, presence of chordee, availability of the prepuce and quality of the urethral plate, in addition to the experience of the surgeon.^4^ The purpose of this study was to determine the surgical and cosmetic outcomes for different types of hypospadias operated in our clinic from January 2000 to December 2017, to identify complications and to suggest new variants of TIPU technique.

## MATERIALS AND METHODS

There were 269 boys (aged between 12 and 43 months) with hypospadias treated at our clinic during the study period from January 2000 to December 2017. The mean age at the operation was 17 months. All of the children underwent primary repair using different types of operation, and they had no history of previous hypospadias repair. The patients were operated under general anesthesia by the same surgical team at our institution. The preoperative meatal site was distal in 179 patients, mid-shaft in 44 and proximal in 46. Penoscrotal cases with severe chordee that required a two-stage repair were excluded from this study. 

The technique used in distal hypospadias was Mathieu in 77 patients, Snodgrass in 28 and tubularized incised plate (TIP)-δ in 74. The TIP-δ method was tubularized incised-plate urethroplasty (TIPU) covered with a penile skin flap created immediately below the distal end of the neourethra as Gardikis *et al.*, from our surgical team described before.^5^^,^^6^ The technique used in mid-shaft hypospadias was tubularized island flap (TIF) in 12 patients, onlay island flap (OIF) in 5 and TIP-ελ in 27. TIP-ελ is the long TIP technique with covering by a second layer from a tunica vaginalis free graft as described by Voges *et al.*^7^

The operative technique for proximal hypospadias was TIF in 15 patients, OIF in 10 and TIP-ελ in 21. In the 35 cases in which the TIP-ελ technique was applied in mid-shaft and proximal hypospadias, we also took small grafts of the tunica vaginalis and the dartos fascia of the prepuce and sent them for histological examination and immunohistochemistry with anti-CD31 monoclonal antibody in order to compare their angiogenic activities. 

Patients were followed up for the maximum time possible, which ranged from 1 to 15 years. The follow-up was performed monthly for the first 6 months, yearly for the next 5 years and then every 2 years for the development of stenosis or assessment of residual chordee. The complications encountered in every technique were assessed statistically using the R software (version 3.4.1). Values were compared using the Chi-Square test. All the statistical tests were performed at the statistical significance level of 5%. We compared all the methods we performed before 2010 with the TIP-δ and TIP-ελ, which are new variants of TIPU that we began to apply in 2010 (Tables 1-3). This study was reviewed and approved by the medical ethical committee of our hospital (Νο. 19357).

**Table 1:** Complications of distal hypospadias operation

**Variable**	Other* **No. (%)**	**TIP-δ** **No. (%)**	**Total** **No. (%)**	
Fistula	5 (4.76)	-	5 (2.79)	
Dehiscence	1 (0.95)	-	1 (0.56)	
Urethra stenosis	-	-	-	
Meatel stenosis	5 (4.76)	2 (2.7)	7 (3.9)	
Total	11 (10.48)	2 (2.7)	13 (7.26)	P: 0.048

*Mathieu technique, *Snodgrass procedure, TIP-δ: Tubularized incised plate-δ

**Table 2:** Complications of mid shaft hypospadias operation

**Variable**	Other* **No. (%)**	** TIP-ελ** **No. (%)**	**Total** **No. (%)**	
Fistula	2 (11.76)	-	2 (4.55)	
Dehiscence	1 (5.88)	-	1 (2.27)	
Urethra stenosis	1 (5.88)	-	1 (2.27)	
Meatel stenosis	-	1 (3.7)	1 (2.27)	
Total	4 (23.53)	1 (3.7)	5 (11.36)	P: 0.043

*TIF: Tubularized island flap, *OIF: Onlay island flap, TIP-ελ: Tubularized incised plate-ελ

**Table 3:** Complications after proximal hypospadias operation

**Variable**	Other* **No. (%)**	** TIP-ελ** **No. (%)**	**Total** **No. (%)**	
Fistula	4 (16)	1 (4.76)	5 (10.87)	
Dehiscence	3 (12)	-	3 (6.52)	
Urethra stenosis	1 (4)	-	1 (2.17)	
Meatel stenosis	-	-	-	
Residual	1 (4)	1 (4.76)	2 (4.35)	
Total	9 (36)	2 (9.52)	11 (23.61)	P: 0.036

*TIF: Tubularized island flap, *OIF: Onlay island flap, TIP-ελ: Tubularized incised plate-ελ

## RESULTS

In distal hypospadias, there were eight complications in the Mathieu procedure (10.39%: Four fistulas, one dehiscence and three meatal stenoses), three in the Snodgrass procedure (10.71%: One fistula and two meatal stenoses) and two in the TIP-δ procedure (2.7%: Two meatal stenoses). The TIP-δ technique produced the smallest number of complications in distal hypospadias (*p*=0.048, Table 1). In mid-shaft hypospadias, the TIF procedure led to three complications (25%: One fistula, one dehiscence and one urethral stenosis), the OIF procedure resulted in one complication (20%: One fistula) and the TIP-ελ procedure had one complication (3.7%: One meatal stenosis). The number of complications in mid-shaft hypospadias was lowest for the TIP-ελ procedure (*p*=0.043, Table 2). 

In proximal hypospadias, there were six complications in the TIF procedure (40%: Three fistulas, two dehiscences and one urethral stenosis), three in the OIF procedure (30%: One fistula, one dehiscence and one residual chordee) and two in the TIP-ελ procedure (9.52%: One fistula and one residual chordee). No instances of testicular atrophy, hematoma or abscess were detected in cases of harvesting a tunica vaginalis graft. The number of complications in proximal hypospadias was lowest for the TIP-ελ technique (*p*=0.036, Table 3). 

Also, we noticed that the free grafts of tunica vaginalis had greater angiogenic activity comparing to the free grafts of dartos fascia of prepuce after immunohistochemistry. The follow up period for most of our patients was between five and seven years. Although most of the parents did not seem to concern after the first year of the operation, because the penis of their child appeared well and they did not notice any difficulty in voiding we asked them to come to all the follow-up appointments in order to detect possible long term complications of our new variants of TIPU. 

The majority of them co-operated with pleasure. Sixteen patients did not come to the follow up appointment again after the first year. Only three of our patients were examined for the last time after nine years, two after eleven, one after thirteen and one in fifteen years after the operation. The use of TIP-δ in distal hypospadias and long TIP-ελ in mid-shaft and proximal hypospadias produced significantly fewer complications than the other surgical methods across all cases of hypospadias (*p*<0.05).

## DISCUSSION

Hypospadias repair even in the hands of the most-experienced pediatric surgeons can be associated with significant complications. Urethrocutaneous fistula, meatal stenosis, urethral stricture, dehiscence and residual chordee are some of the more-frequent complications in the most-severe cases. Since Snodgrass initially reported TIPU for distal hypospadias repair in 1994, this technique has gained widespread acceptance for urethroplasty in both distal and proximal hypospadias.^1^

The probability of complications after TIPU can be reduced by covering the suture line with a well-vascularized tissue flap, since this creates a layer between the two suture lines and probably increases the vascularity to the neourethra.^5^ Our use of a pedicle penile perimeatal-based flap as a second layer of coverage in TIPU resulted in the lowest percentage of complications in cases of distal hypospadias. We experienced no cases of fistula formation or dehiscence, with the two cases of meatal stenosis being treated successfully with dilatation. Furthermore, the overall complication rate with the TIP-δ technique was 2.7%, which is lower than the rates reported recently in the international literature.^8^

Our follow up period gave answer to the limitations of the technique mentioned to our previous article.^5^ We did not notice the formation of any sebaceous inclusion cyst or late ventral curvature in all the children with the TIP-δ technique that came to the seven year follow up appointment. We experienced high complication rates in cases of mid-shaft hypospadias when applying the TIF and OIF procedures, whereas the rate was only 3.7%, when applying TIP-ελ. It was also interesting that we observed no fistulas when using TIP-ελ. 

In more-proximal cases the overall complication rate was higher, as expected, but the TIP-ελ operation is becoming more important due to its lower complication rate (9.52%) compared with the other techniques, that we had applied before 2011 and the outcomes reported recently in other series.^9^ Voghes *et al.* reported in 1990 the first application of free grafts of the tunica vaginalis to close urethrocutaneous fistulas in hypospadias repair, which produced very good results.^7^ Since then, other authors have described the use of tunica vaginalis flaps and grafts in hypospadias surgery.^10^^-^^13^

We preferred to use tunica vaginalis grafts as a second layer in long TIPU techniques because they are easy to harvest and our histological examinations and immunohistochemistry with anti-CD31 monoclonal antibody of some tunica vaginalis tissues of our patients exhibited higher angiogenic activity compared to tissues from the dartos fascia of the prepuce. Also, we did not observe any cases of testicular atrophy, hematoma or abscess in any of the tunica vaginalis grafts that we harvested.

## CONCLUSION

The type of tissue used for neourethral coverage seems to play an important role in the outcome of hypospadias surgery. Reliable results can be obtained by performing neourethral coverage with a perimeatal skin graft in coronal and distal hypospadias and with a tunica vaginalis free graft in mid-shaft and proximal hypospadias. These approaches will reduce the incidence of complications and in particular the appearance of urethrocutaneous fistulas.
